# Hospital Workers Disaster Management and Hospital Nonstructural: A Study in Bandar Abbas, Iran

**DOI:** 10.5539/gjhs.v8n4p221

**Published:** 2015-08-31

**Authors:** Parvin Lakbala

**Affiliations:** 1 Health Information Management Research Center, Hormozgan University of Medical Sciences, Bandar Abbas, Iran; 2 Department of Health Information Technology, Faculty of Para-Medicine, Hormozgan University of Medical Sciences, Bandar Abbas, Iran

**Keywords:** disaster, risk management, hospital, knowledge

## Abstract

**Introduction:**

A devastating earthquake is inevitable in the long term and likely in the near future in Iran. The objective of the study was to assess the knowledge of hospital staff to disaster management system in hospital and to determine nonstructural safety assessment in Shahid Mohammadi hospital in Bandar Abbas city of Iran. This hospital is the main referral hospital in Hormozgan province with a capacity of about 450 beds and the highest patient admissions.

**Methods:**

The cross-sectional study was conducted in 2013 on 200 healthcare workers at Shahid Mohammadi hospital, in the city of Bandar Abbas, Iran. This hospital is the main referral hospital in Hormozgan province and has a capacity of about 450 beds with highest numbers of patient admissions. Questionnaire and checklist used for assessing health workers knowledge and awareness towards disaster management and nonstructural safety this hospital.

**Results:**

This study found that knowledge, awareness, and disaster preparedness of hospital staff need continual reinforcement to improve self efficacy for disaster management. Equipping health care facilities at the time of natural disasters, especially earthquakes are of great importance all over the world, especially in Iran. This requires the national strategies and planning for all health facilities.

**Conclusion:**

It seems due to limitations of hospital beds, insufficient of personnel, and medical equipment, health care providers paid greater attention to this issue. Since this hospital is the only educational public hospital in the province, it is essential to pay much attention to the risk management not only to this hospital but at the national level to health facilities.

## 1. Introduction

Iran is on seismic fault lines. It is exposed to a variety of natural and man-made disasters that have caused considerable damages to the population and infrastructures. Given earthquakes’ potential for devastation, hospitals are among the main critical facilities that should be active continuously after an earthquake in order to provide necessary emergency services. According to the Global Assessment Report on Disaster Reduction (UNISDR, 2009), Iran’s risk class for natural hazards is 8 out of 10. Over the last four decades, these hazards have caused more than 109,000 deaths and 150,000 injuries in Iran (Ardalan et al., 2012). More than 31000 killed in Bam’s earthquake in 2003 and 600 in Zarand’s earthquake in 2005 (Neighboring province of Hormozgan). Bandar Abbas is a port and the capital of Hormozgan Province on the southern coast of Iran, on the Persian Gulf. The city occupies a strategic position on the narrow strait of Hormoz, and it is the location of the main base of the Iranian Navy. Bandar Abbas is also the capital and biggest city of Hormozgan province. At the 2006 census, its population was 367,508, with 89,404 families. Two powerful earthquakes with magnitudes 6 in 2006 and 2008 in the Hormozgan province of southern Iran, near the port city of Bandar Abbas killed totally 10 (7 people in 2008 and 3 people in 2006) peoples and injured more than 50. Adverse impacts of disasters on the Iranian hospitals have been enormous. In the prior earthquake in the Bam all hospitals collapsed. Also in the Zarand earthquake there was hospital chaos for hours due to non-structural damages and absence of staff and in another case the most hospitals were destroyed in the earthquake of the East Azarbaijan, even new constructed hospitals (Ardalan et al., 2014).

Hospital safety from disasters is a challenge in all countries (Sternberg et al., 2004; Krauskopf & Saavedra, 2004; Pan American Health Organization, 2004; Bagaria et al., 2009). Disaster damage to health systems is a human tragedy, results in huge economic losses, deals devastating blows to development goals, and shakes social confidence. Making hospitals and health facilities, safe from disasters is an economic requirement, and also a social, moral and ethical necessity. Health facilities are only truly safe from disasters when they are accessible and functioning, at maximum capacity, immediately after a hazard strike. The hospital safety index is an assessment tool for assessing the structural safety, non-structural safety and functional capacity of hospitals a disaster (WHO, 2008). The non-structural assessment of critical public facilities such as hospital buildings is essential for earthquake disaster adaptation in earthquake prone regions.

The objective of the study was to assess the knowledge of hospital staff to disaster management system in hospital and to determine nonstructural safety assessment at Shahid Mohammadi hospital in Bandar Abbas city of Iran. This hospital is the main referral hospital in Hormozgan province and has a capacity of about 450 beds with highest patient admissions.

## 2. Methods

The cross-sectional study was conducted in 2013 on 200 healthcare workers at Shahid Mohammadi hospital, in the city of Bandar Abbas, Iran. Bandar Abbas (center of Hormozgan province) is the most important port in the south of Iran because of its historical places, cultural, economic, social and political importance. Questionnaire and checklist used for the assessment of health workers knowledge and awareness towards disaster management and nonstructural safety at Shahid Mohammadi hospital.

Stratified sampling was used to choose 300 respondents representing a variety of healthcare professionals and cadres of health workers, including medical doctors, paramedic staff, nurses and other workers. Three hundred questionnaires were distributed and 200 (67%) were returned. Study participants included healthcare personnel working in different departments of the hospital. The questionnaire included 27 questions. Questions were provided and developed following a literature search and review in order to acquire information about workers knowledge and awareness towards disaster management in hospital. This questionnaire included 22 questions regarding the knowledge and awareness of disaster management in hospital and 5 questions regarding staff demographics. The answers about workers knowledge and awareness level were grouped into positive and negative answers, and were used to perform a statistical analysis.

The checklist included 4 parts (external opportunities, thereat, internal weaknesses, and internal strengths) with 33 items regarding non-structural safety of the hospital. For the assessment of non-structural safety of the hospital, researcher observed all wards of the hospital through visiting hospital and also with deep interviewed to the hospital administrator, technical staff, head of hospital departments and the staff who filled checklist.

Questionnaires were distributed among participants who agreed to participate in May 2013, with a follow up visiting to non respondents 2 weeks later. The responses were entered into a spreadsheet and the data entry was verified for accuracy via manual verification. The collected data from questionnaire were analyzed using SPSS v. 12 (SPSS, Chicago, IL, USA) and χ^2^ test, with P < 0.05 considered statistically significant. The percentages and their 95% confidence intervals are presented.

## 3. Results and Discussion

The results of the survey were tabulated and percentile analysis was carried out. This study found that knowledge, awareness and disaster preparedness of hospitals, staff need continual reinforcement to improve self efficacy for disaster management. Three hundred questionnaires were distributed and 200 (67%) were returned. Respondents for this study consisted of 131(65.5%) females and 69 (34.5%) males). Respondents comprised of 14 (7%) doctors, 35 (17.5%) paramedic staff, 108 (54%) nurses and 43 (21.5%) other staff. Years of work experience ranged from 1 to 29 amongst the respondents. The educational status of hospital personnel interviewed were 26 (13%) associate diploma, 130 (65%) bachelor, 31 (15.5%) master and 13 (6.5%) Ph.D, or MD. Respondents awareness of disaster management being conducted in the hospital was 175 (87.5) while 151 (75.5) of participants haven’t aware about the warning system in the hospital.

Hospitals must play a key role in developing disaster preparedness plans, and they need to coordinate efforts with public health systems and appropriate governmental agencies. In USA the joint commission on accreditation of health care organizations (JCAHO) requires hospitals test their emergency plan twice a year, including at least one community-wide drill (JCAHO, 2003). In this study 95 (47.5%) of participants demonstrated that the hospital requires three or more drills in a year.

Current disaster knowledge, skills, and preparedness levels need to be evaluated to guide plans for effective educational programs (Al Khalaileh, 2012). One hundred and ninety two (96%) of participants in this study expressed that the training of disaster management in hospital is useful, although only 34 (17%) of participants had trained in disaster management in hospital.

Trained to cope, retrofitting hospital and assisting with disaster relief teams is so important for the most of participant 163 (81.5). The results showed that there was found to be a statistically significant relationship between the level of education attainments and training in disaster management (χ^2^ = 33.327, d.f. =3, P = 0.000) and the awareness about disaster management (χ^2^ = 12.239, d.f. = 3, P = 0.007) in hospital. This may be from due to the knowledge gained by respondents through training and related courses at the graduate courses. In a survey carried out in Jordan by Al khalaileh and et al. (2006), the findings demonstrated that nurses, interested to learn about the nurses role in disasters, including knowledge and skills. Knowledge, skills, and disaster preparedness need continual reinforcement to improve self efficacy for disaster management (Al Khalaileh, 2012).

The results showed that there found to be a statistically significant relationship between the age work group of attainments and awareness about the warning system in hospital [χ^2^ = 45.523, d.f. =2, P = 0.000) and the awareness about disaster management (χ^2^ = 15.691, d.f. = 2, P = 0.000) in hospital.

The results showed that only fifteen participants (7.5%) mentioned disaster management as the duties of personnel in the hospital. There found to be a statistically significant relationship between the participants awareness about the warning system (χ^2^ = 51.998, d.f. = 3, P = 0.000), training in disaster management in hospital (χ^2^ = 11.170, d.f. = 3, P = 0.011) and disaster management as the duties of personnel in the hospital (χ^2^ = 14.819, d.f. = 3, P = 0.002) with the participants age group. This may be from lack of awareness or lack of systematic training program for personnel toward disaster management in hospitals specially for new personnel. The results of this study showed 166 (83.0%) of participants did not receive any training program in the hospital disaster management, while Compulsory Training of disaster management for all personnel mentioned by the majority of participants in this study 84 (42.0%). This suggests that policy makers in the health field consider the short in-service programs in the field of disaster management in the hospital plans for all personnel.

The most of 190 (95.0%) participants mentioned that the hospital outer space is not suitable for temporary accommodation. One hundred and seven (53.5%) of participants demonstrated the costs of risk management plan in the hospital is not only government or hospital duties but also it is duties of all. Hospital disaster management needs to be tackled and effective management should be a team effort including government and private organizations. It is time for reinforced disaster preparedness plans and drills in the hospital.

The non-structural assessment of critical public facilities such as hospital buildings is essential for earthquake disaster adaptation in earthquake prone regions (Dixit et al., 2014). Hospitals are expected to function as a safe environment during disasters, but many become unusable because of nonstructural damage. The results of study that conducted by Djalali showed the mean non-structural safety index for hospitals of Iran compare to Stockholm is lower and 70% of hospital in Iran were at risk (Djalali et al., 2014).

This study showed despite reconstruction of the hospital in recent years, there are still many internal threats that require attention (Table 2). A study in Taiwan and other studies showed the most hospitals have failed during a disaster because of inappropriate emergency plan and/or non-structural damage (Whitney et al., 2001; Angantyr et al., 2009; Djalali et al., 2014; Yao & Lin, 2000). The studies in Caribbean countries showed moderately safe hospitals (18) while the assessments of Moldova hospitals revealed the better situation (Pisla et al., 2010).

**Table 1:** Participants, awareness toward disaster management

Participant Awareness	Yes (%)	No (%)
Awareness about disaster management in hospital	175 (87.5)	25 (12.5)
Disaster management is duties of personnel in the hospital	15 (7.5)	185 (92.5)
Awareness about the warning system in hospital	49 (24.5)	151(75.5)
Training in disaster management in hospital	34 (17)	166 (83)
Usefulness of training in disaster management in hospital	192 (96)	8 (4)
Exists of Disaster Management Committee in hospital Chart	200 (100)	-

How does disaster management training:		
Compulsory Training for all personnel	84 (42)	116 (58)
Compulsory Training for hospital disaster Committee	10 (5)	190 (95)
Optional Training for hospital disaster Committee	49 (24.5)	151 (75.5)
Trained to cope, retrofitting hospital and assisting with disaster relief teams	163 (81.5)	37 (18.5)

Temporary Accommodation:		
Hospital outer space for temporary accommodation	10 (5)	190 (95)
Place outside the hospitals for temporary accommodation	73 (36.5)	127 (63.5)
Parking lot	6 (3)	194 (97)
Other places	86 (43)	114 (57)

Disaster Costs:		
Direct government costs help in the risk management plan	62 (31)	138 (69)
Hospital income allocation	8 (4)	192 (96)
Assistance of private and government companies	6 (3)	194 (97)
All	107 (53.5)	93 (46.5)

Drill time:		
One time per year	32 (16)	168 (84)
Two times per year	73 (36.5)	127 (63.5)
Three and More times per year	95 (47.5)	105 (52.5)

**Table 2:** Hospital internal strengths and weakness

Internal Weakness	Internal Strengths
Lack of strong organizational homes, in hospital	Disaster management committee in hospital chart
Lack of strong organizational homes, in hospital	Trained and ready personnel in times of crisis
Availability of personnel in the earthquake time	Unit leadership and experienced manager
Lack of water supply	New equipped ambulances
Lack of septic tank	Hospital resistant structures
Lack of several large covered and secure parking to accommodate victims of the earthquake	Hospital escapes stairs
	Hospital automatic fire systems
Lack of emergency exit signs	Hospital Powerful electric generators
Not fixed some of the medical equipment	Hospital diesel fuel storages
Not fixed shelves for medicines and equipment	Hospital standard structural
Telecommunication system	Hospital Standards for equipment installed
	Heating, ventilation and air-conditioning system
	Office and store room furnishings (computers, etc.)
	Medical and laboratory equipment and supplies
	Architectural treatments

Table 3 showed the external opportunities and threats at Shahid Mohammadi hospital. Some non-standard buildings in the area outside of the hospital are a threat for employees and also occupied hospital outer space for temporary accommodation. Protective measures of the hospitals from disasters considered in the strategy of Iran’s health system for disaster risk management in recent year (Ardalan et al., 2012). However, the implementation of these programs should be developed at the national level and comprehensive action plans.

**Table 3:** External opportunities and threats in Shahid Mohammadi hospital

External opportunities	Threats
Adjacent of hospital to Military garrison and bandwidth helicopter	High population density in the hospital area
Great stadium behind the hospital as a safe space for temporary resettlement of victims	Non-standard buildings in hospital area
Specialized health care centers near hospital	Lack of a flyover bridge for pedestrians crossing of highway for access to hospital
Adjacent to the emergency center	
Proximity the hospital to fire station	
Approach roads to hospitals	

## 4. Conclusion

Hospitals are required to serve as a secure environment during disasters, but many become unusable because of nonstructural damage. Equipping health care facilities at the time of natural disasters, especially earthquakes are of great importance all over the universe, especially in developing countries such as Iran. Because Shahid Mohammadi hospital is the only educational public hospital in the city and Hormozgan province, the need to reinforce non-structural hospital, drills and training are indispensable. Implementation of risk management in the hospitals can imrove improve hospital functioning in an earthquake. This requires the national strategies and planning for all health facilities. For developing the disaster preparedness program, putting hospitals, disaster management in assessment and accreditation of the hospitals of Iran may encourage the hospitals to invest on disaster mitigation and preparedness.

It seems due to limitations of hospital beds, insufficient of personnel, and medical equipment, health care providers paid greater attention to this matter. But with regards this hospital is only educational general hospital in the province, greater attention to the risk management is essentialis necessary to focus more on disaster management in health facilities.
